# MicroRNA-126 Inhibits SOX2 Expression and Contributes to Gastric Carcinogenesis

**DOI:** 10.1371/journal.pone.0016617

**Published:** 2011-01-27

**Authors:** Takeshi Otsubo, Yoshimitsu Akiyama, Yutaka Hashimoto, Shu Shimada, Kentaro Goto, Yasuhito Yuasa

**Affiliations:** Department of Molecular Oncology, Graduate School of Medical and Dental Sciences, Tokyo Medical and Dental University, Tokyo, Japan; Emory Unviersity, United States of America

## Abstract

**Background:**

SRY (sex-determining region Y)-box 2 (SOX2) is a crucial transcription factor for the maintenance of embryonic stem cell pluripotency and the determination of cell fate. Previously, we demonstrated that SOX2 plays important roles in growth inhibition through cell cycle arrest and apoptosis, and that SOX2 expression is frequently down-regulated in gastric cancers. However, the mechanisms underlying loss of SOX2 expression and its target genes involved in gastric carcinogenesis remain largely unknown. Here, we assessed whether microRNAs (miRNAs) regulate SOX2 expression in gastric cancers. Furthermore, we attempted to find downstream target genes of SOX2 contributing to gastric carcinogenesis.

**Methodology/Principal Findings:**

We performed *in silico* analysis and focused on miRNA-126 (miR-126) as a potential SOX2 regulator. Gain- and loss-of function experiments and luciferase assays revealed that miR-126 inhibited SOX2 expression by targeting two binding sites in the 3′-untranslated region (3′-UTR) of *SOX2* mRNA in multiple cell lines. In addition, miR-126 was highly expressed in some cultured and primary gastric cancer cells with low SOX2 protein levels. Furthermore, exogenous miR-126 over-expression as well as siRNA-mediated knockdown of SOX2 significantly enhanced the anchorage-dependent and -independent growth of gastric cancer cell lines. We next performed microarray analysis after SOX2 over-expression in a gastric cancer cell line, and found that expression of the *placenta-specific 1* (*PLAC1*) gene was significantly down-regulated by SOX2 over-expression. siRNA- and miR-126-mediated SOX2 knockdown experiments revealed that miR-126 positively regulated *PLAC1* expression through suppression of SOX2 expression in gastric cancer cells.

**Conclusions:**

Taken together, our results indicate that miR-126 is a novel miRNA that targets SOX2, and *PLAC1* may be a novel downstream target gene of SOX2 in gastric cancer cells. These findings suggest that aberrant over-expression of miR-126 and consequent SOX2 down-regulation may contribute to gastric carcinogenesis.

## Introduction

The *SOX2* gene encodes a member of the SRY-related HMG-box (SOX) family of transcription factors involved in the regulation of embryonic development and in the determination of cell fate [1], [2], [3]. In particular, it is well known that SOX2 plays important roles in maintenance of embryonic stem (ES) cell self-renewal and pluripotency [4], [5]. Among adult tissues, SOX2 is expressed in the brain, retina, tongue, lung, esophagus and stomach, and plays crucial roles in the differentiation and morphogenesis of these organs [6], [7], [8]. We previously reported that SOX2 mRNA and protein were expressed in normal gastric mucosae, but frequently down-regulated in human gastric cancer tissues and cell lines, some of which are due to aberrant DNA methylation [9], [10]. We further revealed that SOX2 plays important roles in growth inhibition through cell cycle arrest and apoptosis, indicating that SOX2 may have tumor-suppressive functions in gastric cancer cells [10]. However, the downstream target genes of SOX2 involved in gastric carcinogenesis remain largely unknown.

MicroRNAs (miRNAs) are small, approximately 22-nucleotide, noncoding RNAs that regulate the expression of hundreds of genes by targeting their mRNAs posttranscriptionally [11]. miRNAs bind to the partially complementary target sites in 3′-untranslated regions (3′-UTRs) of mRNAs, inducing direct mRNA degradation or translational inhibition [11]. To date, it has been reported that the miRNA expression profiles differ between in normal tissues and derived tumors, including gastric cancer, and many miRNAs can act as tumor suppressors or oncogenes [12], [13], [14]. Recently, it was reported that miRNA-134 and miRNA-145 repress SOX2 expression by targeting its coding region in mouse ES cells and the 3′-UTR in human ES cells, respectively [15], [16]. However, there have been no reports on miRNA(s) that can regulate SOX2 expression in human gastric cancer.

In the initial step of this study, we performed immunohistochemical analysis of the SOX2 protein in human gastric cancer tissues, in which the DNA methylation statuses of *SOX2* had already been examined [10], and found that a certain number of SOX2 expression-negative cases did not show DNA hypermethylation, leading us to the idea that there is another mechanism underlying SOX2 down-regulation. Accordingly, in this study, we aimed to find miRNAs that target SOX2 expression in human gastric cancers. We found that miRNA-126 (miR-126) repressed SOX2 expression by targeting its 3′-UTR, and then performed functional analyses of miR-126 in gastric cancer cells. To further clarify the importance of miR-126-mediated SOX2 down-regulation in gastric carcinogenesis, we attempted to identify downstream target genes of SOX2 in gastric cancer cells.

## Results

### The *SOX2* 3′-UTR is a predicted target of miRNA-126 and -522

**Figure 1:**
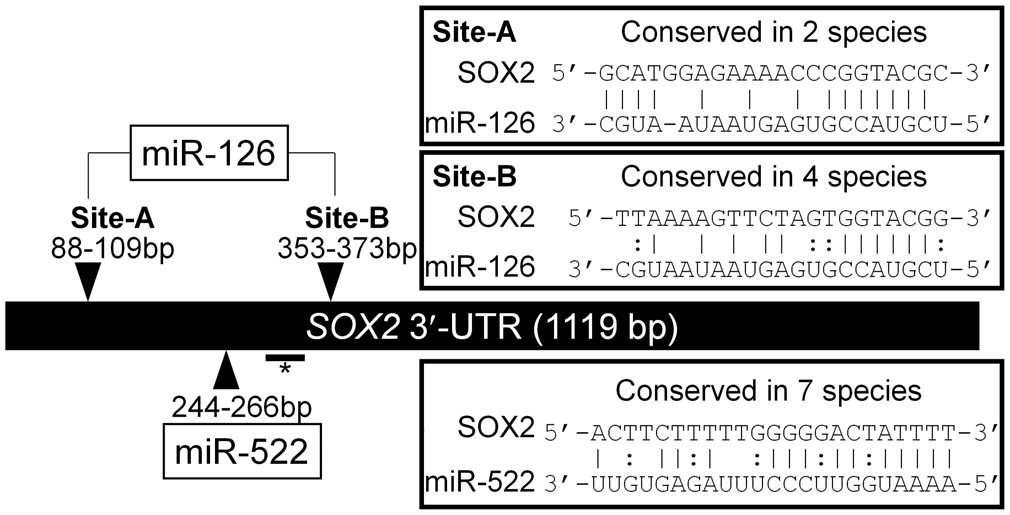
Schematic diagrams of predicted target sites of miR-126 and miR-522 in the *SOX2* 3′-UTR. The predicted binding sites of miR-126 and miR-522 are indicated (arrowheads) in the *SOX2* 3′-UTR (1119 bp). The first nucleotide after the stop codon of *SOX2* is defined as “1”, and the start- and end-positions of the complementary sequence between SOX2 and miRNAs are indicated above or beneath the arrowheads. *The horizontal bar below the *SOX2* 3′-UTR indicates the region targeted by the SOX2 siRNA. Sequence alignments of miR-126 and miR-522 with their corresponding potential target sites in the *SOX2* 3′-UTR are presented in each rectangle. The conservation status among species of the predicted binding sites is also indicated in each rectangle.

In order to find novel miRNAs that regulate SOX2 expression in gastric cancer, we performed computational analysis using a miRNA target database, MicroCosm Targets (formerly miRBase Targets), and tried to identify miRNAs that target the *SOX2* 3′-UTR according to the following criteria. Considering the position, number, and sequence conservation of miRNA target sites among species, we selected two miRNAs, miR-126 and miR-522, as potential miRNAs targeting the *SOX2* 3′-UTR (Figure 1). miR-126 has two predictive target sites, which are both near the stop codon of the *SOX2* open reading frame (ORF) in the 3′-UTR, whereas the predicted target site of miR-522 is highly conserved in seven species and also located near the *SOX2* ORF stop codon in the 3′-UTR (Figure 1).

### miR-126 inhibits SOX2 expression in multiple cell lines

**Figure 2:**
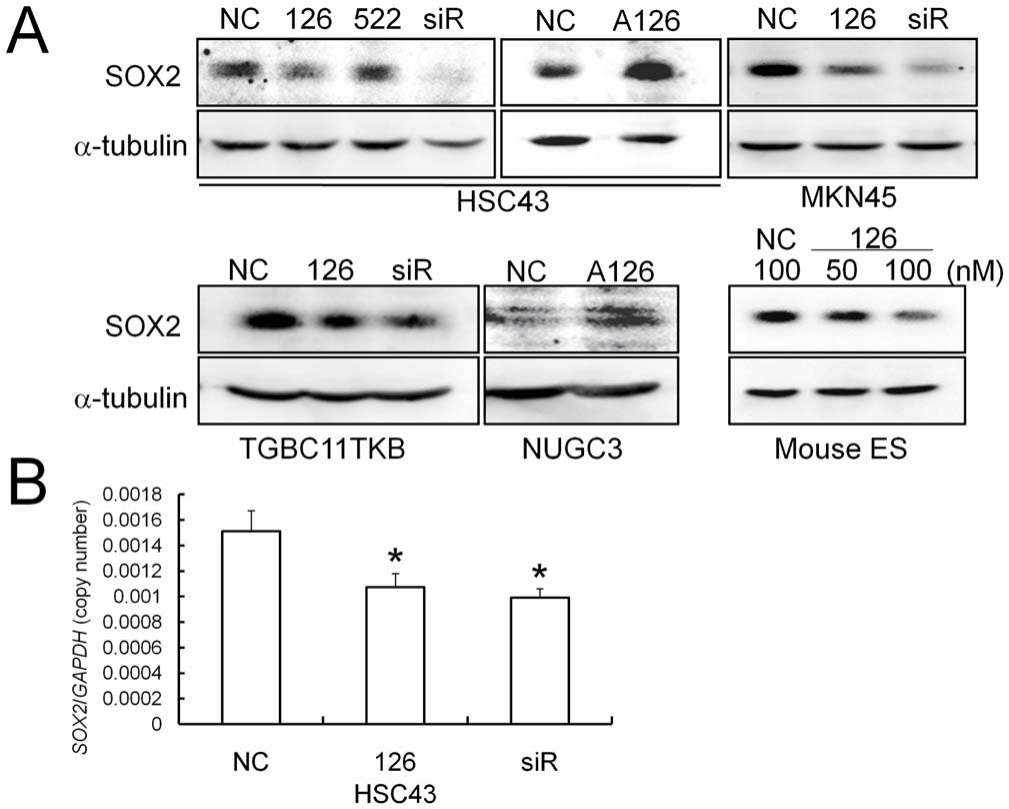
Effects of miR-126 and miR-522 on SOX2 expression. (**A**) Western blot analysis of SOX2 protein expression after transfection of a negative control oligonucleotide (NC), Pre-miR-126 (126), Pre-miR-522 (522), SOX2 siRNA (siR), and Anti-miR-126 (A126) in the indicated gastric cancer cell lines and mouse ES cells. The final concentrations were 50 nM for Pre-miRNAs and siRNA, and 100 nM for Anti-miR-126, respectively. α-tubulin expression was used as a protein loading control. (**B**) Quantitative real-time RT-PCR analysis of *SOX2* mRNA expression after transfection of the negative control, Pre-miR-126 and SOX2 siRNA into HSC43 cells. The expression levels were normalized against internal *GAPDH* expression. The assays were performed in triplicate, and the bars indicate s.d. *P<0.05.

To validate the results of computational analysis, we examined whether or not miR-126 and miR-522 can repress the expression level of the endogenous SOX2 protein in SOX2-expression-positive gastric cancer cell lines. As shown in the upper panel of Figure 2A, transfection of the miR-126 mimic molecule (Pre-miR-126) as well as SOX2 siRNA markedly reduced the endogenous SOX2 protein level compared with a non-specific negative control oligonucleotide (NC) in HSC43 cells, but the miR-522 mimic molecule (Pre-miR-522) did not.

To generally evaluate the possibility that miR-126 inhibits SOX2 expression, we transfected Pre-miR-126 and Anti-miR-126 inhibitor (Anti-miR-126) into multiple gastric cancer cell lines. We used the following cell lines for transient transfection experiments: MKN45 (SOX2 positive; miR-126 intermediate) and TGBC11TKB (SOX2 positive; miR-126 negative) for Pre-miR-126 transfection; and HSC43 (SOX2 positive; miR-126 positive) and NUGC3 (SOX2 very low; miR-126 positive) for Anti-miR-126 transfection (Table 1). Remarkable reductions of the SOX2 protein level were observed in Pre-miR-126-transfected MKN45 and TGBC11TKB cell lines (Figure 2A). Conversely, Anti-miR-126 transfection up-regulated the SOX2 protein levels in both the HSC43 and NUGC3 cell lines (Figure 2A), indicating that not only exogenous Pre-miR-126 but also endogenous miR-126 can regulate SOX2 protein levels in gastric cancer cells. We also performed quantitative real-time RT-PCR analysis of *SOX2* mRNA expression, and found that exogenous miR-126 modestly but significantly suppressed the *SOX2* mRNA level in HSC43 cells (Figure 2B).

**Table 1:** Expression of SOX2 and miR-126 in gastric cancer cell lines.

	SOX2^a^		
Cell line	Protein	mRNA	miR-126^b^
TGBC11TKB	+++	+++	-
HSC43	++	+++	+++
MKN45	++	+++	+
KATOIII	+	+++	-
AGS	+	+++	-
HSC44PE	±	++	±
NUGC3	±	+	++
GCIY	-	±	++
NUGC4	-	-	++
HSC58	-	-	+++

a Classification of band intensity: -, completely invisible; ±, faintly visible; +, visible; ++, clearly visible; +++, strongly visible.

b miR-126 expression was measured with TaqMan and calculated by the delta-delta Ct method using RNU6B as an internal control. The intensity of miR-126 to RNU6B was defined as follows: -, <0.05; ±, 0.05∼0.09; +, 0.1∼0.79; ++, 0.8∼2.0; +++, >2.0.

Because the SOX2 protein is known to be abundantly expressed in ES cells, we examined whether or not miR-126 inhibits SOX2 protein expression in a mouse ES cell line (SOX2 positive; miR-126 negative). Interestingly, exogenous miR-126 transfection dose-dependently decreased the SOX2 protein level in the mouse ES cells (Figure 2A), suggesting that miR-126 represses SOX2 expression in various species and cell lineages.

### miR-126 directly targets the *SOX2* 3′-UTR through two predicted binding sites

**Figure 3:**
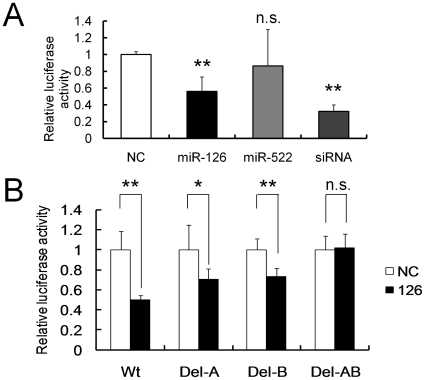
Interaction between miR-126 and its binding sites in the *SOX2* 3′-UTR. (**A**) Dual luciferase assay with the pGL4-*SOX2* 3′-UTR (1050 bp) reporter vector (Wt). 30 nM Pre-miRNAs or SOX2 siRNA, which targets the *SOX2* 3′-UTR, was co-transfected with 10 ng of the indicated reporter vector into HEK293T cells. (**B**) Dual luciferase assay with cotransfection of 10 ng of the reporter vectors containing the wild type *SOX2* 3′-UTR (Wt), single deletion mutant A (Del-A), single deletion mutant B (Del-B), or double deletion mutant AB (Del-AB), and 30 nM negative control or Pre-miR-126 in HEK293T cells. The assays were performed in triplicate, and the bars indicate s.d. *P<0.05; **P<0.01; n.s., not significant.

To determine whether or not the predicted target sites for the miRNAs in the 3′-UTR of *SOX2* mRNA are responsible for the SOX2 down-regulation, we performed luciferase reporter assays with a vector containing the *SOX2* 3′-UTR downstream of the luciferase reporter gene. As shown in Figure 3A, significant repression of luciferase activities were observed in HEK293T cells co-transfected with the pGL4-*SOX2* 3′-UTR vector and Pre-miR-126 or siRNA that targets the *SOX2* 3′-UTR (Figure 1). On the other hand, Pre-miR-522 had no significant effect on the luciferase activity of the pGL4-*SOX2* 3′-UTR vector compared with NC (Figure 3A). These results, combined with those of Western blot analysis, indicate that miR-126 is a more potential candidate miRNA that represses SOX2 expression in gastric cancer than miR-522.

To examine the direct interaction of miR-126 with the potential target sites in the *SOX2* 3′-UTR, we carried out luciferase reporter assays with the deletion mutant vector as to the putative miR-126 target sites. As shown in Figure 1, miR-126 has two predicted binding sites, A and B, in the 3′-UTR of *SOX2* mRNA. We therefore performed luciferase assays with the wild type pGL4-*SOX2* 3′-UTR vector (Wt), the vectors with each predicted miR-126 target site deleted, A (Del-A), B (Del-B), or both sites, AB (Del-AB). Intriguingly, each single deletion mutant vector exhibited a low inhibitory effect on luciferase activity compared with the Wt vector after Pre-miR-126 co-transfection (Figure 3B). Moreover, the double deletion mutant vector, Del-AB, showed complete reversal of the inhibitory effect of the Pre-miR-126 co-transfection (Figure 3B), indicating that miR-126 directly inhibits SOX2 expression by targeting the two binding sites in the 3′-UTR of *SOX2* mRNA independently.

### The inverse correlation between miR-126 and SOX2 expression in some cultured and primary gastric cancer cells

To assess the relationship between miR-126 and SOX2 expression in gastric cancers, we initially examined SOX2 mRNA and protein levels by RT-PCR and Western blot analysis, respectively, in 10 gastric cancer cell lines without DNA methylation of *SOX2* (Table 1) [10]. Five of the 10 cell lines showed low or undetectable levels of the SOX2 protein, whereas three of them exhibited a low *SOX2* mRNA level (Table 1). We subsequently examined miR-126 expression by TaqMan real-time PCR analysis in these 10 gastric cancer cell lines. Four (NUGC3, GCIY, NUGC4 and HSC58) of the 10 cell lines, whose SOX2 mRNA and protein levels were low, exhibited relatively high expression of miR-126, whereas the other cell lines, except for HSC43 cells, exhibited relatively low expression of miR-126 and a high *SOX2* mRNA level (Table 1). These data indicate that the miR-126 expression level is mostly opposite to the SOX2 mRNA and protein levels in gastric cancer cell lines.

**Figure 4:**
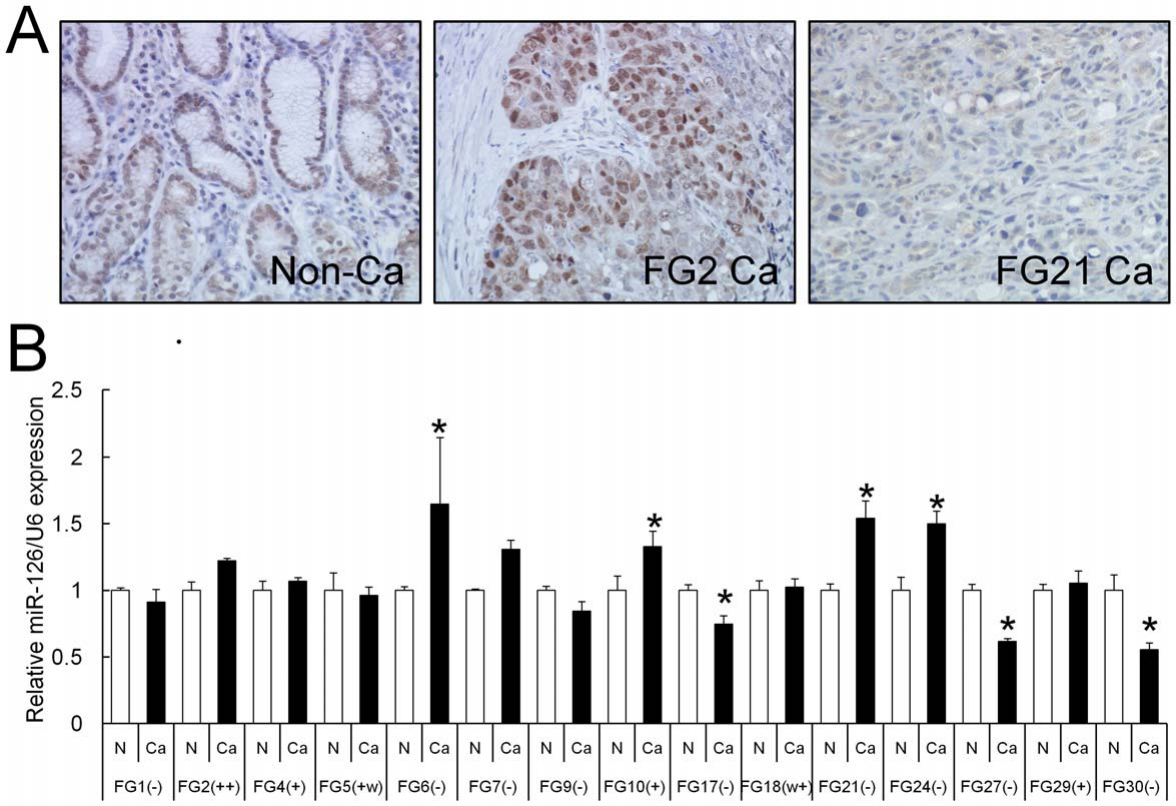
Expression of SOX2 and miR-126 in human gastric cancer tissues. (**A**) Representative immunohistochemical staining of SOX2 protein in non-cancerous mucosa (Non-Ca) and gastric cancers (FG2 Ca and FG21 Ca). Original magnification: ×400. (**B**) Quantitative TaqMan real-time PCR analysis for miR-126 was carried out by using the 15 human gastric cancer tissues (filled bars) and paired non-cancerous tissues (open bars). The expression levels of cancer tissues were independently compared to those of paired non-cancerous tissues, which are normalized to 1, and the bars indicate s.d. *P<0.05. The intensities of SOX2 expression were indicated beneath each case by x-axis. The expression levels were determined by the following criteria: “++” for 10% or more cancer cells were strongly stained; “+” for 10% or more cancer cells were stained; “w+” for less than 10% cancer cells were weakly stained; “–” for almost all cells were negatively stained.

To further compare the expression pattern of miR-126 with that of SOX2 in primary gastric cancers, we initially examined the expression levels of SOX2 protein in 15 primary gastric cancer tissue samples without DNA methylation of *SOX2* by immunohistochemistry. We found that almost all non-cancerous mucosae showed SOX2-positive signal only in the cell nuclei within the neck of the gastric glands (Figure 4A), whereas nine of the 15 cases exhibited low or undetectable levels of the SOX2 protein compared with the paired non-cancerous mucosae (Figure 4).

Next, total RNA was isolated from these 15 gastric cancers and paired non-cancerous tissues, and the miR-126 expression levels were determined by TaqMan real-time PCR analysis. Four of the 15 cases exhibited significantly high levels of miR-126 expression, whereas three of them did low miR-126 levels in comparison with the adjacent non-cancerous mucosae (Figure 4B). Among the miR-126-up-regulated cases, three (FG6, FG21 and FG24) exhibited lower levels of SOX2 protein than paired non-cancerous mucosae (Figure 4B), suggesting that high levels of miR-126 expression contribute to low levels of SOX2 protein at least in some primary gastric cancers. There was no significant correlation between the miR-126 expression and sex, age, depth of tumor invasion or histological type (data not shown).

### miR-126 enhanced anchorage-dependent and -independent growth of gastric cancer cells

**Figure 5:**
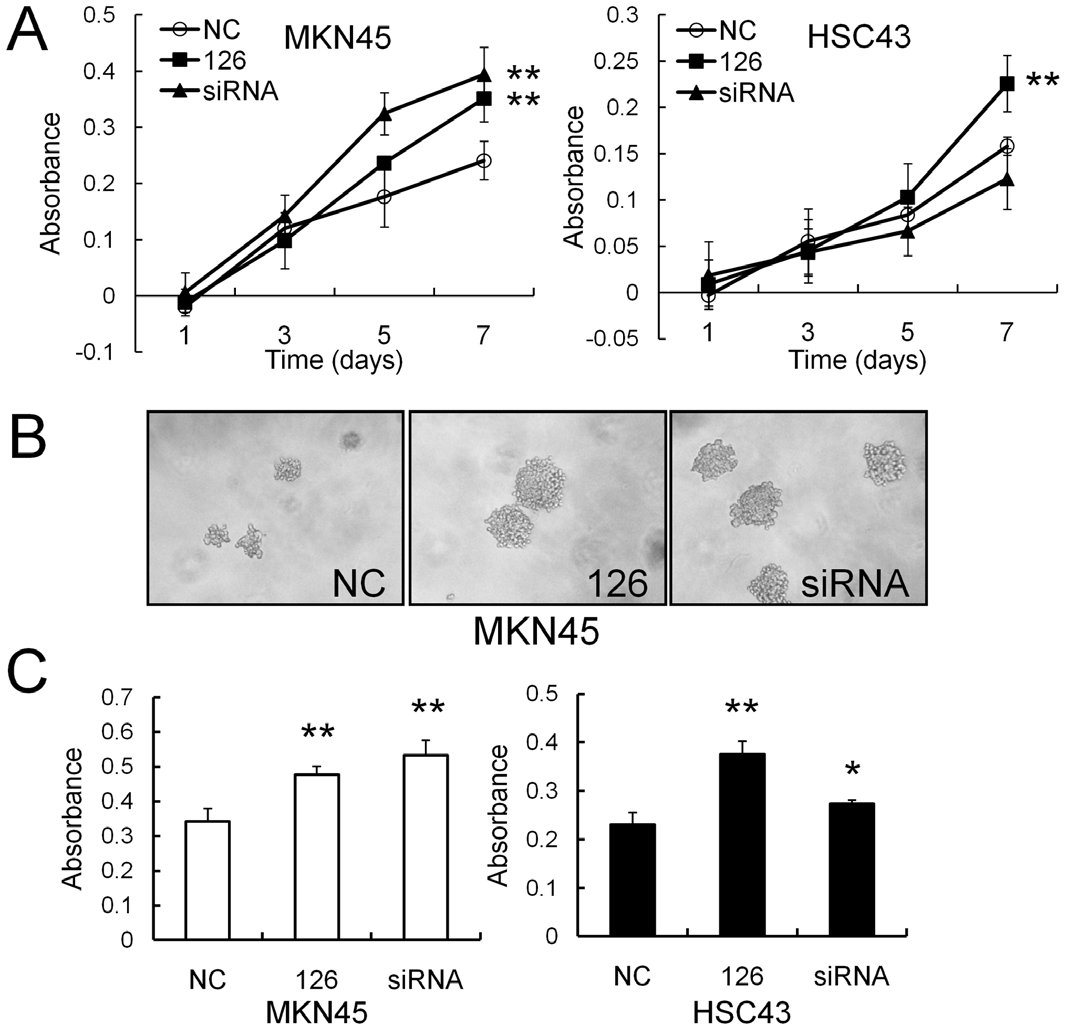
Effects of miR-126 expression on anchorage-dependent and -independent cell growth. (**A**) *In vitro* cell proliferation assays after SOX2 knockdown by Pre-miR-126 or siRNA in gastric cancer cell lines. The number of viable cells was determined with a Cell Counting Kit-8 on days 1, 3, 5 and 7 after plating. (**B**) Representative phase contrast microphotographs of the colonies of MKN45 cells in soft agar at 9 days after transfection of the negative control, Pre-miR-126 or SOX2 siRNA. Original magnification: ×100. (**C**) Soft agar colony formation assays for measurement of the anchorage-independent growth of gastric cancer cell lines. The vertical axis (Absorbance) indicates the relative number of colony-forming cells, which was determined by the colorimetric assay method. The assays were performed in quadruplicate, and the bars indicate s.d. *P<0.05; **P<0.01.

We next evaluated the effect of miR-126 on tumor cell growth. We initially examined the proliferation rates of SOX2-expression positive gastric cancer cell lines, MKN45 and HSC43, after transient transfection of Pre-miR-126. As shown in Figure 5A, the Pre-miR-126-transfected-MKN45 and HSC43 cells exhibited significant growth advantages compared with the control NC-transfected-cells. Moreover, the SOX2 siRNA-transfected-MKN45 cells, but not HSC43 cells, significantly increased proliferation compared with the control cells (Figure 5A), suggesting that miR-126-mediated growth stimulation may occur in a SOX2-dependent manner, at least in MKN45 cells.

To determine the role of miR-126 in gastric tumorigenesis, we next carried out soft agar colony formation assays of gastric cancer cell lines after Pre-miR-126 transfection (Figure 5B and C). As shown in Figure 5B, the Pre-miR-126- and SOX2 siRNA-transfected-MKN45 cells formed larger colonies than the NC-transfected cells in soft agar at 9 days after transfection. We then performed soft agar colony formation assays using a CytoSelect™ 96-Well In Vitro Tumor Sensitivity Assay Kit, which can be used to count the colony-forming cells by means of a colorimetric method, such as the WST-8 assay, making the assays quick and accurate. Exogenous miR-126 over-expression as well as SOX2 siRNA transfection significantly enhanced the anchorage-independent colony formation of MKN45 and HSC43 cells compared with the control cells at 9 to 10 days after transfection, respectively (Figure 5C). These results suggest that miR-126 may promote the tumorigenicity of gastric cancer cells through suppression of SOX2 expression.

### miR-126 controls novel SOX2 target genes in gastric cancer cells

**Figure 6:**
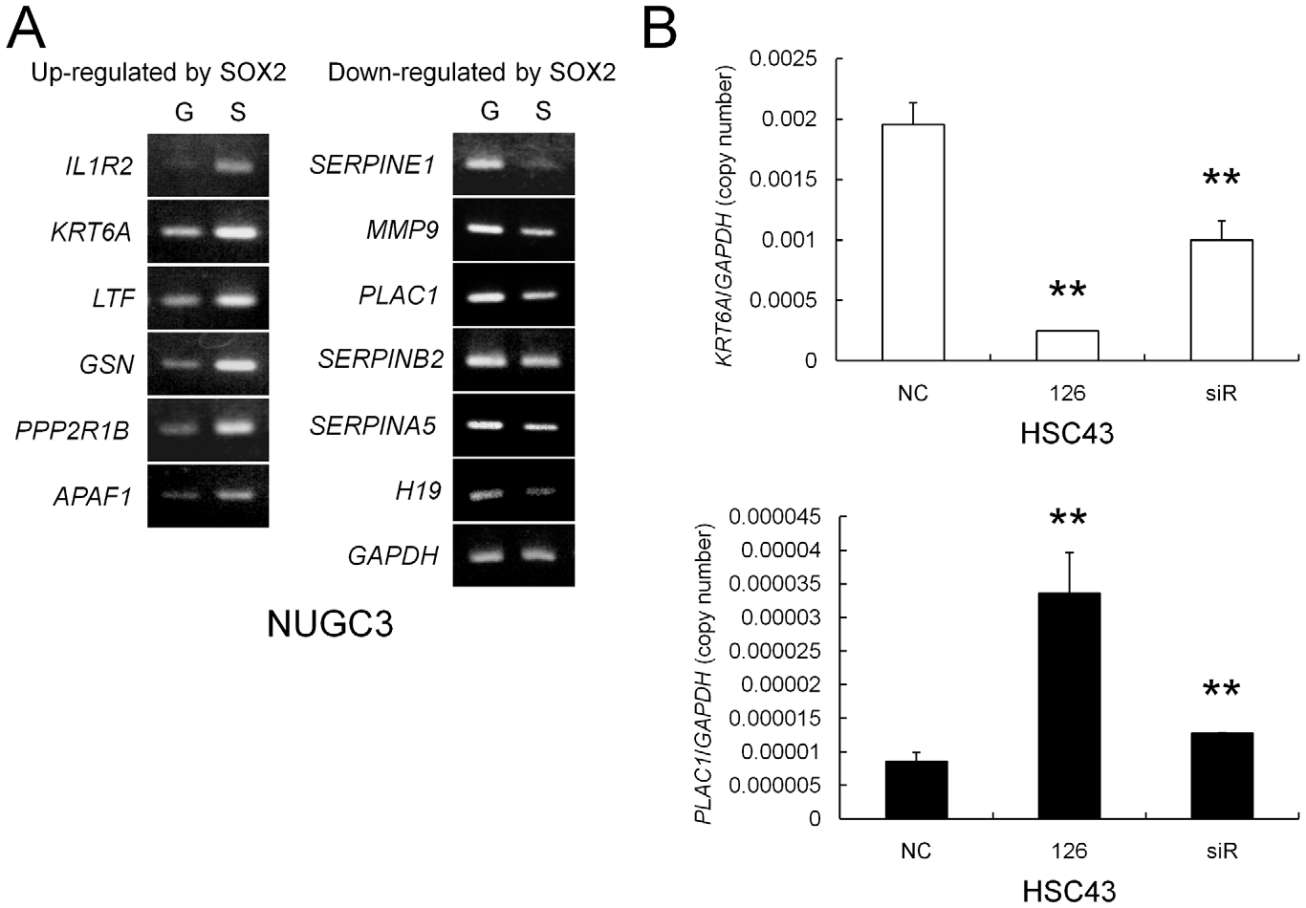
Expression changes of predicted SOX2 target genes. (**A**) Changes in gene expression after adenovirus-mediated ectopic SOX2 over-expression in NUGC3 cells. RT-PCR analysis was performed to validate the cDNA microarray results for Ad-GFP-infected (G) and Ad-SOX2-infected NUGC3 cells (S). *GAPDH* expression was used as an internal loading control. (**B**) Quantitative real-time RT-PCR analysis of the *KRT6A* and *PLAC1* mRNA expression levels after SOX2 knockdown by Pre-miR-126 or siRNA in HSC43 cells. The expression levels were normalized against internal *GAPDH* expression. The assays were performed in triplicate, and the bars indicate s.d. **P<0.01.

To further understand the potential effects of miR-126-mediated SOX2 down-regulation on the gene expression change in gastric cancer cells, we first attempted to identify candidate downstream target genes of SOX2. We transiently expressed exogenous SOX2 in NUGC3 cells by using an adenovirus system, and changes in expression were determined by cDNA microarray analysis (GEO accession No. GSE23589). Among 41,174 probes, 366 known genes were up-regulated (>2.0-fold) and 369 known genes were down-regulated (<0.5-fold) by SOX2-over-expression in NUGC3 cells compared with in control GFP-over-expressing cells (Table S1). Representative microarray results are summarized in Table 2, and we found the significant up-regulation of exogenous SOX2 (>20.20-fold), supporting the validity of this experiment. Intriguingly, there were many cancer-related genes that could be novel downstream targets of SOX2 (for example, *LTF*, *PPP2R1B*, *TGFBR2*, *SERPINE1*, *MMP9*, *HMGA1*, *SOX9* and *PLAC1*), and squamous cell differentiation markers *KRT6E* and *KRT6C*, whose amino acid sequences are highly conserved among the KRT6 family members and virtually identical to one of the known SOX2 downstream genes, *KRT6A* (Table 2 and Table S1) [17]. We validated the microarray results by RT-PCR analysis in NUGC3 cells after SOX2 over-expression, and representative results are shown in Figure 6A. Most of these genes also showed changes in their expression after SOX2 over-expression at least in one more gastric cancer cell line among the two to three cell lines we investigated (data not shown).

**Table 2:** Representative results of microarray analysis by SOX2 over-expression.

Gene symbol	Gene name	Fold change
SPP1	secreted phosphoprotein 1 (osteopontin)	63.54
SOX2	SRY (sex determining region Y)-box 2	20.20
IL1R2	interleukin 1 receptor, type II	12.53
KRT6E	keratin 6C (virtually identical to KRT6A)	10.52
LTF	lactotransferrin	7.85
GSN	gelsolin (amyloidosis, Finnish type)	5.97
KRT6C	keratin 6C (virtually identical to KRT6A)	7.07
PPP2R1B	protein phosphatase 2 regulatory subunit A beta isoform	3.86
KLK10	kallikrein-related peptidase 10	3.24
EPB41L1	erythrocyte membrane protein band 4.1-like 1	2.81
PRKAR2B	protein kinase, cAMP-dependent, regulatory, type II, beta	2.75
TGFBR2	transforming growth factor, beta receptor II (70/80kDa)	2.73
SOCS1	suppressor of cytokine signaling 1	2.57
APAF1	apoptotic peptidase activating factor 1	2.22
IL7R	interleukin 7 receptor	0.13
SERPINE1	serpin peptidase inhibitor, clade E, member 1 (PAI-1)	0.15
MMP9	matrix metallopeptidase 9	0.27
PLAC1	placenta-specific 1	0.28
PDZK1IP1	PDZK1 interacting protein 1	0.29
SERPINB2	serpin peptidase inhibitor, clade B, member 2 (PAI-2)	0.32
HMGA1	high mobility group AT-hook 1	0.37
SERPINA5	serpin peptidase inhibitor, clade A, member 5 (PAI-3)	0.38
H19	H19, imprinted maternally expressed untranslated mRNA	0.42
SOX9	SRY (sex determining region Y)-box 9	0.47

To determine the target genes of SOX2 controlled by miR-126 in gastric cancer cells, we next performed SOX2 knockdown experiments and further screened for candidate target genes. SOX2 knockdown by Pre-miR-126 and siRNA was confirmed by Western blot analysis in SOX2-expression-positive gastric cancer cell lines MKN45 and HSC43 (Figure 2A), and the subsequent expression changes of the putative SOX2 downstream target genes were preliminarily analyzed by RT-PCR in these cell lines, and then by quantitative real time RT-PCR in HSC43 cells. Among over 20 cancer-related genes we investigated, only two showed changes in expression after SOX2 knockdown (data not shown). First, differentiation marker *KRT6A* expression, which was up-regulated by SOX2 over-expression, was significantly down-regulated by Pre-miR-126 as well as SOX2 siRNA transfection in HSC43 cells (Figure 6B). Second, placenta- and tumor-specific *PLAC1* expression, which was down-regulated by SOX2 over-expression, was significantly up-regulated by SOX2 knockdown with Pre-miR-126 and siRNA in HSC43 cells (Figure 6B). These results indicate that miR-126 can control *KRT6A* and *PLAC1* expression by down-regulating SOX2 expression in gastric cancer cells, and these genes might be downstream target genes of SOX2 contributing to gastric carcinogenesis.

## Discussion

We previously reported that SOX2 expression was frequently down-regulated in human gastric carcinoma tissues (about half of the total cases), some of which was due to aberrant DNA methylation (about 16% of the total cases) [10]. Therefore, the mechanisms underlying loss of SOX2 expression have not yet been defined in more than half of the cases. In this study, we demonstrated that miR-126 decreased the SOX2 mRNA and protein expression levels in gastric cancer cell lines. In addition, we found that miR-126 expression was inversely correlated with SOX2 expression in certain cultured and primary gastric cancer cells without DNA methylation of *SOX2*, indicating that aberrant miR-126 expression may be a novel mechanism underlying SOX2 down-regulation in gastric cancer. Furthermore, Pre-miR-126 over-expression promoted anchorage-dependent and -independent growth of gastric cancer cells *in vitro*, and increased oncogenic *PLAC1* expression in a gastric cancer cell line. These findings suggest that miR-126 may be an oncogenic miRNA that controls SOX2 expression in gastric cancer cells. Besides gastric cancer, Pre-miR-126 over-expression reduced the SOX2 protein level in mouse ES cells, suggesting that miR-126 may generally control SOX2 expression, at least in two species (human and mouse) and various cell lineages, including ES cells.

MiR-126 is known as an endothelium-specific miRNA, and has been reported to promote angiogenesis by targeting *SPRED1* and *PIK3R2*, which normally inhibit VEGF signaling [18], [19], [20]. Moreover, it has been reported that miR-126 inhibits apoptosis of acute myeloid leukemia (AML) cells and enhanced the colony-forming ability of mouse bone marrow progenitor cells through targeting Polo-like kinase 2 (*PLK2*), a tumor suppressor [21]. On the contrary, miR-126 has also been reported to be a tumor suppressive miRNA, inhibiting tumor cell growth through targeting p85beta in colon cancer cell lines, and targeting IRS-1 in HEK293 and MCF-7 cells, respectively [22], [23]. Although it is controversial as to whether miR-126 is a tumor suppressive or oncogenic miRNA, at least in the present study, we demonstrated that miR-126 acts as an oncogene by targeting SOX2 in gastric cancer cells. These functional differences in oncogenesis might be considered to be a “lineage-dependency model for cancer”, that is, developmentally important genes also have crucial roles during tumor progression in lineage-specific manners [24]. However, further studies are needed to clarify the biological roles of miR-126 in gastric carcinogenesis and other tissues.

The initial computational analysis indicated that both miR-126 and miR-522 are candidate miRNAs that target the *SOX2* 3′-UTR. However, miRNA over-expression experiments showed that Pre-miR-126 but not Pre-miR-522 reduced SOX2 protein levels and *SOX2* 3′-UTR luciferase activity. Moreover, loss-of-function experiments and reporter assays involving deletion mutants of *SOX2* 3′-UTR luciferase vectors revealed that miR-126 directly targets the 3′-UTR of *SOX2*. This difference between miR-126 and miR-522 as to SOX2 regulation is likely to be due to the following reasons. First, the 5′ region, which is called the miRNA “seed” sequence (nucleotides 2–7), of miR-126 completely matches the 3′-UTR of *SOX2*, whereas miR-522 does not. It is well known that perfect “seed” pairing is required for both target site recognition and repression of the target transcript [25], [26]. Second, miR-126 has two binding sites in the 3′-UTR of *SOX2* mRNA, but miR-522 has only one. It has been reported that when a miRNA has multiple binding sites in the 3′-UTR of its target gene, the binding sites could be simultaneously targeted by the miRNA [27], [28]. These findings combined with our present data suggest that the presence of multiple complementary target sites and perfect matches between these ones and miRNA “seed” region are good indicators for finding functional miRNAs.

In this study, miR-126 expression was found to be relatively high in SOX2-expression-negative gastric cancer cell lines, and was aberrantly up-regulated in some primary gastric cancer cases compared with the paired non-cancerous mucosae. However, the mechanism underlying this aberrant miR-126 expression in gastric cancer remains to be elucidated. It was previously reported that the over-expression of miR-126 in a kind of AML, core-binding factor (CBF)-AML, is associated with partial demethylation of the CpG island but not with amplification or mutation of the genomic locus [21], [29]. In fact, we observed that some gastric cancer cell lines exhibited restored miR-126 expression after treatment with a demethylating agent, 5-aza-2′-deoxycytidine (data not shown). These findings indicate that miR-126 expression may be epigenetically regulated in gastric cancer cells.

We performed cDNA microarray analysis to identify the downstream target genes of SOX2 in gastric cancer cells, and found that many tumor-associated genes exhibited significant changes in expression after SOX2-overexpression (e.g., *LTF*, *PPP2R1B*, *TGFBR2*, *SERPINE1*, *MMP9*, *HMGA1* and *SOX9*). Furthermore, most of them also exhibited changes in their expression after SOX2-overexpression, at least in two gastric cancer cell lines. These results indicate that SOX2 might regulate the expression of these tumor-associated genes, thereby contributing to gastric carcinogenesis. However, we could not observe any significant changes in expression of these genes after SOX2 knockdown by Pre-miR-126 or siRNA, at least in the cell lines we tested. There are some possible reasons for this discrepancy. First, it has been reported that a different expression level of SOX2 switches the regulation of target gene expression from up- to down-regulation, or vice versa [30], [31]. In this study, the expression levels of SOX2 were quite different among the cell lines that were used for the over-expression and knockdown experiments. Second, it is well known that stem cell transcription factors, such as SOX2, OCT3/4 and Nanog, cooperatively interact with their target genes' promoters and control their gene expression, being so-called “transcriptional cofactors”. These expression differences and/or transcriptional cofactors might also be critical for control of expression of the downstream target genes of SOX2 in gastric cancer cells, and further studies are necessary to clarify the roles of SOX2 in the regulation of its target genes.

Expression of *KRT6A* and *PLAC1* was significantly changed by both SOX2 over-expression and knockdown, suggesting that SOX2 is the critical regulatory factor for these two genes in gastric cancer cells. *KRT6A* is a member of the cytokeratin gene family, and recently it was reported that ectopic SOX2 over-expression up-regulated the *KRT6A* mRNA level in a lung adenocarcinoma cell line [17]. In the present study, we demonstrated the possibility that *KRT6A* expression is also positively regulated by SOX2 in gastric cells, but the role of *KRT6A* in gastric carcinogenesis remains unclear. Thus, further investigations are necessary to elucidate the roles of *KRT6A* in gastric carcinogenesis.

On the other hand, *PLAC1*, a recently described X-linked gene exhibiting expression restricted to the placenta, is also expressed in a wide variety of human cancers, including gastric cancer [32], [33]. Koslowski *et al.* reported that siRNA-mediated knockdown of PLAC1 decreased cell motility, migration and invasion, and induced G1-S cell cycle arrest with nearly complete abrogation of proliferation in breast cancer cell lines [34]. In this study, we demonstrated that SOX2 negatively regulates *PLAC1* expression in gastric cancer cell lines, and propose a novel hypothesis that miR-126 inhibits SOX2 expression and consequent changes in the expression of some SOX2 target genes, such as *PLAC1*, thereby contributing to gastric carcinogenesis.

In conclusion, for the first time, we demonstrated that miR-126 is a novel oncogenic miRNA, which targets SOX2, and that downstream pro-oncogenic target genes of SOX2, such as *PLAC1*, may contribute to gastric carcinogenesis. These findings have important implications for not only explaining the loss of SOX2 expression in gastric cancers, but also for understanding the transcriptional regulatory mechanisms of SOX2 in other various cell lineages, such as ES cells. Taken together, our findings may lead to new diagnostic and therapeutic approaches for gastric cancer, and provide new insights into the transcriptional regulation of SOX2.

## Materials and Methods

### Ethics Statement

Written informed consent was obtained from all subjects, and the study was approved by the Ethics Committee of Tokyo Medical and Dental University.

### Cell lines and tissue samples

We used 10 human gastric cancer cell lines (HSC43, MKN45, TGBC11TKB, NUGC3, KATOIII, AGS, HSC44PE, GCIY, NUGC4 and HSC58) and one human embryonic kidney cell line (HEK293T) in this study, as described previously [10], [35]. All the cell lines were cultured in appropriate medium. Mouse ES cell line BL6 was obtained from Dr. Hirobumi Teraoka (Tokyo Medical and Dental University Medical Research Institute, Japan), and was cultured as described previously [36]. A total of 16 primary gastric carcinoma tissue samples and corresponding non-cancerous gastric mucosae were obtained, as described previously [10].

### miRNA mimic and inhibitor transfection

Gastric cancer cells were transfected with Precursor Molecules mimicking miR-126 (Pre-miR-126), miR-522 (Pre-miR-522) (Applied Biosystems, Foster City, CA), SOX2 siRNA (sense, 5′-GGAAUGGACCUUGUAUAGAUC-3′; and anti-sense, 5′-UCUAUACAAGGUCCAUUCCCC-3′, Sigma-Aldrich, St. Louis, MO), anti-miR inhibitor miR-126 (Anti-miR-126) (Dharmacon, Lafayette, CO), or scrambled sequence miRNA (Pre-miR-NC) (Pre-miR Negative Control #1, Applied Biosystems) to give a final concentration of 10 to 100 nmol/L (nM) by using MicroPorator MP-100 (Digital BioTechnology, Seoul, Korea), according to the manufacturer's instructions. At 24–72 h after transfection, cells were harvested for Western blot or RT-PCR analyses.

### Western blot

Western blot analyses were performed as described previously [10]. The primary antibodies used were rabbit anti-SOX2 (1∶1000: Cell Signaling Technology, Danvers, MA) and mouse anti-α-tubulin (1∶200; Santa Cruz Biotechnology, CA). The secondary antibodies used were alkaline phosphatase-conjugated anti-mouse IgG or anti-rabbit IgG (1∶2000; Bio-Rad Laboratories, Hercules, CA). Blots were developed with Immun-Star™ AP Substrate (Bio-Rad Laboratories). We used α-tubulin as an internal protein loading control, and the band intensities were defined as described in the footnote of Table 1 when 100 µg of protein was loaded per lane.

### RT-PCR and quantitative real-time RT-PCR

Total RNA was extracted by using Trizol reagent (Invitrogen, Carlsbad, CA) and treated with DNA-free™ (Applied Biosystems). RT-PCR and quantitative real-time RT-PCR were performed as described previously [10]. The primer sequences used for all genes are shown in Table S2. For semi-quantitative RT-PCR, *GAPDH* expression was used as an internal loading control, and the band intensities were defined as described in the footnote of Table 1 under the conditions of 35 PCR cycles.

### Dual luciferase reporter assay

The 3′-UTR oligonucleotide of *SOX2*, a 1050 bp fragment containing the last 36 bps of the *SOX2* coding region and the putative target sites of miR-126 and miR-522, was amplified by PCR with the following primers: sense, 5′-GCGCTCTAGAGCCATTAACGGCACACTGCC-3′; and anti-sense, 5′-GGCCTCTAGATACATGGATTCTCGGCAGAC-3′. Luciferase constructs were obtained by ligating the wild type 3′-UTR oligonucleotide of *SOX2* (Wt) or nucleotides with the miR-126 target sites deleted (Del-A, -B or -AB) into the *Xba*I site of the pGL4.13 (*luc2*/SV40) *firefly* luciferase reporter vector (Promega, Madison, WI). HEK293T cells were co-transfected using HiPerFect (QIAGEN, Hilden, Germany) with 10 ng of the pGL4.13 vector containing or not containing the 3′-UTR sequence (for normalization of the non-specific effects on pGL4.13-3′-UTR vector of miRNAs), 4 ng of the pGL4.74 (*hRluc*/TK) *renilla* luciferase control vector (for normalization of the transfection efficiency), and 30 nM Pre-miR-126, Pre-miR-522, SOX2 siRNA, or Pre-miR-NC. Luciferase activity was measured 24 h after transfection using a Dual-Luciferase Reporter Assay System (Promega). Relative luciferase activity was calculated by normalizing the *firefly* luminescence as to the *renilla* luminescence.

### Immunohistochemistory

Paraffin-embedded tissue samples were sectioned, deparaffinized, and then pretreated by autoclaving in 10 mM citric acid buffer for 15 min to retrieve antigenicity. After the peroxidase activity had been blocked with 3% H_2_O_2_-methanol for 15 min, the sections were incubated with 10% normal goat serum in PBS to block nonspecific protein binding, followed by incubation with primary antibody against SOX2 (1∶300; Millipore) at 4°C overnight. Then, the sections were incubated with horseradish peroxidase-labeled goat anti-mouse-rabbit antibody (Dako, Carpinteria, CA) for 30 min at room temperature, and the signal was amplified and visualized with diaminobenzidine-chromogen, followed by counterstaining with hematoxylin. Expression was considered to be “positive” when 10% or more cancer cells were stained.

### Quantitative real-time RT-PCR of miRNA

Total RNA was extracted by using Trizol reagent (Invitrogen) and then treated with DNA-free™ (Applied Biosystems) for cell lines. On the other hand, paraffin-embedded tissue samples were sectioned into 10 µm-thick, deparaffinized under RNase-free condition, and then total RNA was extracted by using RecoverAll™ Total Nucleic Acid Isolation Kit (Applied Biosystems, Foster City, CA) according to manufacturer's instructions. Quantitative real-time RT-PCR of miRNA was carried out using a TaqMan Reverse Transcription Kit (Applied Biosystems), TaqMan MicroRNA Assays (Applied Biosystems), and a LightCycler TaqMan Master (Roche Diagnostics, Mannheim, Germany), according to the manufacturers' instructions. The expression levels of miRNA were calculated by the delta-delta Ct method using RNU6B as an internal control.

### Cell proliferation and soft agar colony formation assays

We transfected Pre-miR-126, SOX2 siRNA and Pre-miR-NC into HSC43 and MKN45 cell lines to give a final concentration of 50 nM by using MicroPorator MP-100. After 48 hours, the transfected cells were trypsinized, counted and replated in quadruplicate on 96-well plates (5×10^2^ cells for HSC43, 2.5×10^2^ cells for MKN45 per well). Cell proliferation was evaluated on days 1, 3, 5 and 7 after replating by determining the number of cells with a Cell Counting Kit-8 (Dojindo, Kumamoto, Japan), according to the manufacturer's instructions.

For soft agar colony formation assays, we used a CytoSelect™ 96-Well In Vitro Tumor Sensitivity Assay Kit (Cell BioLabs, Inc., San Diego, CA), according to the manufacturer's instructions. Briefly, the transfected cells, as described above, were trypsinized, counted and plated in quadruplicate on 96-well plates with Agar Matrix Layer (2×10^3^ cells for HSC43, 1×10^3^ cells for MKN45 per well). After incubating the cells for 7 to 8 days at 37°C and 5% CO_2_, the soft agar in each well was solubilized, and viable cells, that is, colony-forming cells, were measured with Cell Counting Kit-8 (Dojindo).

### Microarray analysis

Adenovirus (Ad)-SOX2 and control Ad-GFP vectors were generated as described previously [10], and used to infect NUGC3 cells at the optimum MOI (infectious units/cell) of 20. At 72 h after infection, total RNA was extracted by using Trizol reagent (Invitrogen) and then treated with DNA-*free*™ (Applied Biosystems). cDNA microarray analysis was conducted by DNA Chip Research Inc. (Kanagawa, Japan) with Whole Human Genome oligo DNA arrays (Agilent Technologies, Santa Clara, CA). The microarray data is Minimum Information About a Microarray Experiment (MIAME) compliant and has been deposited in a MIAME compliant database, Gene Expression Omnibus (GEO). The GEO accession number is GSE23589.

## Supporting Information

Table S1 List of genes up- (>2.0-fold) and down-regulated (<0.5-fold) by SOX2 over-expression.
(XLS)

Table S2 Sequences of RT-PCR primers used in this study.
(XLS)
